# Normobaric Oxygen (NBO) Therapy Reduces Cerebral Ischemia/Reperfusion Injury through Inhibition of Early Autophagy

**DOI:** 10.1155/2021/7041290

**Published:** 2021-06-30

**Authors:** Meng Wang, Xiaokun Geng, Chaitu Dandu, Radhika Patel, Yuchuan Ding

**Affiliations:** ^1^China-America Institute of Neuroscience, Beijing Luhe Hospital, Capital Medical University, Beijing, China; ^2^Department of Neurology, Beijing Luhe Hospital, Capital Medical University, Beijing, China; ^3^Department of Neurosurgery, Wayne State University School of Medicine, Detroit, MI, USA; ^4^Drexel University College of Medicine, Philadelphia, PA, USA; ^5^Department of Research and Development Center, John D. Dingell VA Medical Center, Detroit, MI, USA

## Abstract

**Objectives:**

Normobaric oxygen (NBO) therapy has great clinical potential in the treatment of ischemic stroke, but its underlying mechanism is unknown. Our study aimed to investigate the role of autophagy during the application of NBO on cerebral ischemia/reperfusion injury.

**Methods:**

Male Sprague Dawley rats received 2 hours of middle cerebral artery occlusion (MCAO), followed by 2, 6, or 24 hours of reperfusion. At the beginning of reperfusion, rats were randomly given NBO (95% O_2_) or room air (21% O_2_) for 2 hours. In some animals, 3-methyladenine (3-MA, autophagy inhibitor) was administered 10 minutes before reperfusion. The severity of the ischemic injury was determined by infarct volume, neurological deficit, and apoptotic cell death. Western blotting was used to determine the protein expression of autophagy and apoptosis, while mRNA expression of apoptotic molecules was detected by real-time PCR.

**Results:**

NBO treatment after ischemia/reperfusion significantly decreased infarct volume and neurobehavioral defects. The increased expression of the autophagy markers, including microtubule-associated protein 1A light chain 3 (LC3) and Beclin 1, after ischemia/reperfusion was reversed by NBO, while promoting Sequestosome 1 (p62/SQSTM1) expression. In addition, NBO reduced cerebral apoptosis in association with alleviated BAX expression and increased BCL-2 expression. 3-MA reduced autophagy and apoptotic death but did not further improve NBO-attenuated ischemic damage.

**Conclusion:**

NBO induced remarkable neuroprotection from ischemic injury, which was correlated with blocked autophagy activity.

## 1. Introduction

Ischemic stroke mortality has decreased over the past decade due to intravenous thrombolysis and mechanical thrombectomy restoring blood supply to ischemic neurons [1–3]. The recovery of blood flow after ischemia can occasionally have a paradoxical effect by causing brain edema or hemorrhagic transformation through oxidative stress, complement activation, and destruction of the blood-brain barrier (BBB) [4, 5]. There have been developments in reperfusion combined with neuroprotective approaches to improve stroke prognosis [6, 7], but only a few have made significant advances.

Oxygen inhalation has emerged as an effective neuroprotective strategy for restoring blood flow to an infarcted brain by increasing oxygen supply and improving microcirculation to the ischemic penumbra—a viable yet nonfunctioning area surrounding an ischemic core [8]. Normobaric oxygen (NBO) therapy has drawn attention to itself as a prospect in alleviating hypoxic brain injury because it is widely available and easily administered. Previous studies with rodents have shown that short durations of NBO in the early stages of ischemia onset could extend the reperfusion time window and protect the blood-brain barrier [9–11]. Our very recent randomized controlled clinical trial showed that recanalization combined with high-flow oxygen inhalation improved functional outcomes and reduced mortality in patients with anterior circulation infarction [12]. Thus far, the anti-ischemia effect of NBO has proven to improve tissue oxygenation, increase cerebral blood flow, protect the blood-brain barrier (BBB), and decrease cell apoptosis [10, 13–15]. Even though NBO is believed to have neuroprotective effects, its clinical application has remained stagnant, and research on its potential mechanisms continues.

Autophagy is a self-degrading process that maintains homeostasis by removing superfluous organelles and damaged proteins through a lysosome pathway [16, 17]. Both *in vitro* and *in vivo* experiments provide evidence that autophagy is activated after ischemia [18–21]. It is reported that hyperbaric oxygen (HBO) regulates ischemia-induced autophagy and apoptosis and produces a neuroprotective effect by promoting the recovery of neural function [22]. In the model of repeated cerebral ischemia/reperfusion, HBO treatment attenuated excessive autophagy in the prefrontal cortex [23]. On the contrary, HBO preconditioning could increase the formation of autophagosomes, a sign of autophagy activation. An intracerebroventricular inhibition of autophagy with 3-methyladenine (3-MA) canceled out the neuroprotection of HBO on ischemic injury [24]. Further studies showed that HBO preconditioning promoted protective autophagy through ischemia-induced upregulation of cystatin C [25]. Taken together, autophagy plays an important role in oxygen therapy after ischemic stroke. However, whether NBO affects neuron autophagy in a MCAO model remains to be determined. We speculated that the neuroprotective effect of NBO after ischemia/reperfusion is associated with autophagy.

In this study, autophagy protein microtubule-associated protein 1A/1B-Light chain 3 (LC3), p62/SQSTM1, and Beclin 1 were detected in ischemic stroke, and the key role that autophagy plays in ischemic stroke after NBO treatment was demonstrated by using NBO together with an intracerebroventricular injection of 3-MA.

## 2. Materials and Methods

### 2.1. Animals

Our experiments were institutionally approved by the Ethics Committee for Animal Experimentation of Capital Medical University (Beijing, China), in accordance with the National Institutes of Health (NIH, 92 Bethesda, MD, USA) Guide for the Care and Use of Laboratory Animals (the ethical number 20180901). Sprague Dawley adult rats (male, 280–320 g) were kept in a separate cage where they could get food and water. The rats were randomly assigned to 5 groups: (1) Sham without transient middle artery cerebral occlusion (tMCAO) (*n* = 6), (2) 2-hour tMCAO + 2-hour reperfusion (*n* = 24), (3) 2-hour tMCAO + 6-hour reperfusion (*n* = 24), (4) 2-hour tMCAO + 24-hour reperfusion (*n* = 24), and (5) 2-hour tMCAO + 6-hour reperfusion + 3-MA (rats in this group underwent the same procedure as the third group but were treated with an intracerebroventricular (i.c.v.) injection of 400 nmol 3-MA 10 minutes before reperfusion) (*n* = 24). Each treatment group (*n* = 6) was randomly and equally assigned to the treatment with NBO or room air.

### 2.2. Transient MCAO

Animals fasted 12 hours before the operation. All operations were performed under anesthesia. Rats were placed in a chamber filled with a 1% to 3% isoflurane in a 70 : 30 mixture of nitrous oxide/oxygen and maintained with 1% isoflurane. The right common carotid artery (CCA), the right internal carotid artery (ICA), and the right external carotid artery (ECA) were separated by a longitudinal midline incision in the rats' necks. An intraluminal filament was inserted through the right CCA into the right ICA and pulled out after 2 hours to induce transient middle artery occlusion. Body temperature (rectal temperature), blood pH, pCO_2_, pO_2_, blood glucose, and mean arterial pressure (MAP) were regularly monitored during the operation process.

### 2.3. NBO Inhalation

Rats were placed in an atmospheric oxygen chamber at the beginning of reperfusion. Oxygen inhalation concentration was 95%, and the oxygen flow rate was controlled at 2 L/min. The rats in all 5 treatment groups received the same amount of NBO therapy for 2 hours. The carbon dioxide emitted was removed by adding soda lime (Sigma, USA). In contrast, the room air groups were exposed to a normal atmosphere during the whole experiment.

### 2.4. Drug Application

The anesthetized rats were placed on a stereospecific instrument. A 4.0 mm deep hole was drilled into the rats' skulls 1.4 mm laterally and 0.8 mm posteriorly from the bregma for intracerebral ventricle (i.c.v.) injection as described [26]. Rats were subjected to 400 nmol 3-MA (Sigma, M9281) 10 minutes before reperfusion. 3-MA was dissolved in saline and heated to 60–70°C before injection. The determination of the drug dosage and the method of usage depend on the previously published literature [27, 28].

### 2.5. Infarct Volume Measurement

Rat brains in NBO or room air groups were cut into 2 mm thick coronal slices after 2, 6, and 24 hours of reperfusion. Brain tissue was stained with 2,3,5-triphenyltetrazole sodium chloride (TTC, 125 Sigma, USA). To eliminate calculation error due to edema, we represented the infarct area indirectly by calculating the percentage of infarct volume relative to the total ipsilateral brain area [29].

### 2.6. Evaluation of Neurobehavioral Deficits

The 5-scoring system was used to evaluate the neurobehavioral score in a blind manner [30]. Briefly, neurological function was measured on a 5-point scale from 0 to 4, with 0 representing normal and 4 representing the greatest neurological damage. The neurobehavioral assessment of rats was performed after 2, 6, and 24 hours of reperfusion. All rats had normal nerve function prior to MCAO. A higher score indicates more severe neurological damage.

### 2.7. Real-Time Reverse Transcription-Polymerase Chain Reaction (RT-PCR)

The right ischemic brain hemispheres were removed after 2, 6, and 24 hours of reperfusion and frozen at −80°C for later use. This study used a sensitive real-time polymerase chain reaction technique to detect mRNA expression. With the application of TRIzol reagent (Life Technologies, Carlsbad, CA, USA), the total RNA was isolated from homogenized brain tissue. Then, the total RNA was converted into cDNA through the High Capacity cDNA Reverse Transcription Kit (Applied Biosystems, Foster City, CA, USA), which was quantized to detect gene expression by Prism 7500 real-time PCR (Applied Biosystems). The relative expressions of BCL-2 and Bax were detected in all groups, and GAPDH was used as a control gene. The primers for real-time polymerase chain reaction (PCR) analysis are listed as follows: BCL-2: forward: 5′-AGC ATG CGA CCT CTG TTT GA-3′, reverse: 5′-TCA CTT GTG GCC CAG GTA TG-3′; BAX: forward: 5′-CAC GTC TGC GGG GAG TCA C-3′, reverse: 5′-TTC TTG GTG GAT GCG TCC TG-3′; GAPDH: forward: 5′-GAT GGT GAA GGT CGG TGT GA-3′, reverse: 5′-AGA TGG TGA TGG GTT TCC CG-3′.

### 2.8. Western Blot

Proteins (40 *μ*g) extracted from the ischemic brain (including cortex and striatum) were separated onto SDS-polyacrylamide membrane and probed with primary antibody (rabbit anti-LC3 (1 : 1000; Bimake, China), rabbit anti-Beclin 1 (1 : 1000; Cell Signaling Technology, USA), mouse anti-p62 (1 : 2000; Abcam, USA), rabbit anti-BCL-2 (1 : 1000; Cell Signaling Technology, USA), and rabbit anti-BAX (1 : 2000; Cell Signaling Technology, USA)) overnight at 4°C. Secondary antibodies, used for all primary antibodies, were goat anti-rabbit and anti-mouse IgG-horseradish peroxidase (Santa Cruz) for 1 hour at room temperature after three washes. A monoclonal antibody against *β*-actin (1 : 1000; Abcam, USA) was used as a control in protein gels. To quantify protein expression, western blot images were analyzed by ImageJ for relative grayscale (National Institutes of Health, Bethesda, MD, USA).

### 2.9. Apoptotic Cell Death with ELISA

The cytoplasmic histone-associated DNA fragments in ischemia brains were detected by photometric enzyme immunoassay (Rat cell apoptosis factor (Fas) ELISA kit; Shanghai Enzyme-linked Biotechnology Co., Ltd, China). In short, the right ischemic tissue, containing striatum and frontoparietal cortex, was homogenized and then centrifuged for 30 min at 12,000 r/min to collect the supernatant. Each group was then tested according to the manufacturer's instructions. To reduce the error, each measurement was made in duplicate. Finally, a multimode detector (Beckmandx-880) was used to detect the absorbance at 405 nm.

### 2.10. Statistical Analysis

All data was determined as mean ± SE. GraphPad 155 Prism v7.0 (GraphPad Software, San Diego, CA) was used for statistical analysis. Differences among multiple groups were assessed using a one-way analysis of variance (ANOVA). Post hoc comparison was detected using the least significant difference test with the significance level at *p* < 0.05.

## 3. Results

### 3.1. Physiological Parameters

There were no significant differences in blood pH, blood glucose, PaCO_2_, and mean arterial pressure (MAP) between the sham, NBO, and room air groups (data not shown). Body temperature (rectal area) was maintained at about 37°C. The NBO group showed an increase in PaO_2_ to 400 mmHg (*p* < 0.05, data not shown).

### 3.2. Ischemic Infarct Volume and Neurological Deficit

**Figure 1:**
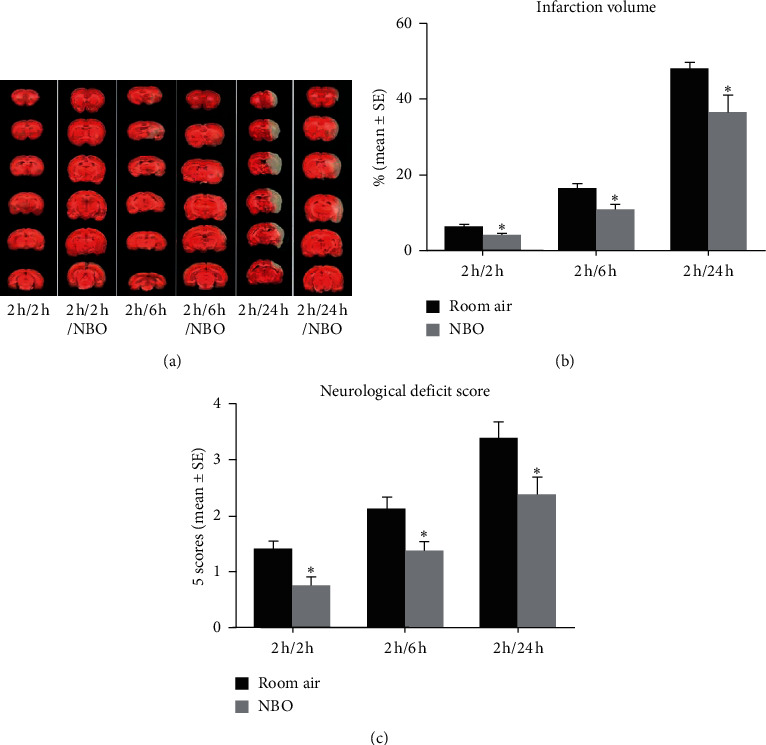
NBO decreased ischemia-induced brain infarction and improved nerve defect. (a, b) Brain sections by TTC staining: red for normal tissue and white for infarcted tissue. After 2 (*p* < 0.05), 6 (*p* < 0.05), and 24 hours (*p* < 0.05) of reperfusion, NBO significantly reduced infarct volume compared to room air. (c) Neurological deficit was determined by the Long-ga 5-scoring system. Compared to the room air group, NBO improved neurological outcomes after 2 (*p* < 0.05), 6 (*p* < 0.05), and 24 hours (*p* < 0.05) of reperfusion. ^*∗*^*p* < 0.05 vs. room air.

In all ischemia/reperfusion groups, infarct volumes were increased. The NBO group had significantly decreased brain infarction at different reperfusion points than the room air group (*p* < 0.05; Figures 1(a) and 1(b)). The neurological deficit score was significantly reduced in the NBO group after 2, 6, and 24 hours of reperfusion compared to the room air group (*p* < 0.05; Figure 1(c)). These data demonstrate that after ischemia, NBO can provide quick neuroprotection with only two hours of reperfusion (see Figure 1).

### 3.3. Expression of Autophagy Proteins

**Figure 2:**
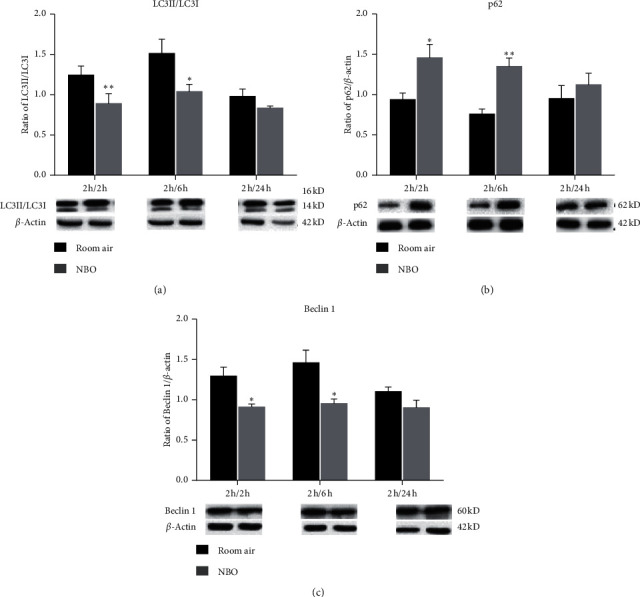
NBO inhibited autophagy activation after early reperfusion by regulating the expression of autophagy-related proteins. (a–c) Western blot and quantitative evaluation of LC3, p62, and Beclin 1 expression. Compared with the room air group, NBO significantly inhibited the increase of LC3II/I and Beclin 1 at early reperfusion (*p* < 0.05, *p* < 0.01). NBO induced p62 expression at 2 and 6 hours (*p* < 0.05; *p* < 0.01) of reperfusion. ^*∗*^*p* < 0.05 and ^*∗∗*^*p* < 0.01 vs. room air.

To determine the effect of NBO on autophagy, we examined the expression of autophagic markers LC3, Beclin 1, and p62 after 2, 6, and 24 hours of reperfusion. Compared to the room air group, a significant reduction in the ratio of LC3II/LC3I and expression of Beclin 1 was seen in the NBO group at 2 and 6 hours of reperfusion (*p* < 0.05, *p* < 0.01; Figures 2(a) and 2(c)). The expression of p62 was significantly increased at 2 and 6 hours of reperfusion compared to the room air group (*p* < 0.05 and *p* < 0.01; Figure 2(b)). After 24 hours of ischemia/reperfusion, NBO did not significantly alter the expression of autophagic markers compared with the room air. These results suggest that NBO mainly mitigates autophagy in the early stages of reperfusion (see Figure 2).

### 3.4. Apoptotic Cell Death and Molecules

**Figure 3:**
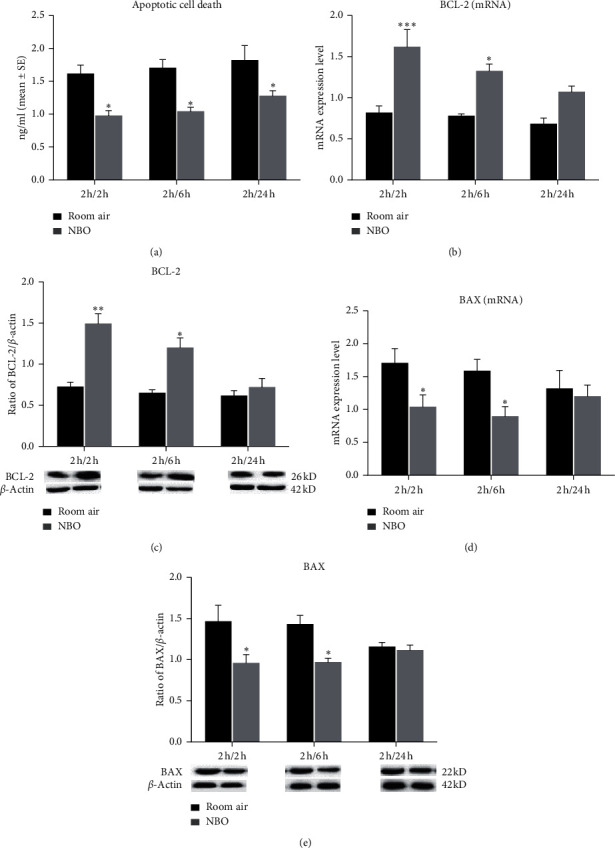
The elevation of apoptosis after ischemia/reperfusion was reduced by NBO. (a) Quantitative evaluation of cell death level measured by ELISA kit. NBO reduced apoptosis after reperfusion in comparison with room air (*p* < 0.05). (b, c) Compared to room air intervention, NBO significantly upregulated mRNA and protein expression of BCL-2 at 2 (*p* < 0.01, *p* < 0.001) and 6 hours (*p* < 0.05) of reperfusion. (d, e) NBO largely decreased mRNA and protein expression of BAX at 2 (*p* < 0.05) and 6 hours (*p* < 0.05) of reperfusion versus room air. ^*∗*^*p* < 0.05, ^*∗∗*^*p* < 0.01, and ^*∗∗∗*^*p* < 0.001 vs. room air.

Apoptotic cell death was determined by histone-associated DNA fragments and through analyzing mRNA and protein levels of antiapoptotic BCL-2 and proapoptotic BAX. NBO significantly reduced apoptotic cell death compared to the room air group at 2, 6, and 24 hours of reperfusion (*p* < 0.05; Figure 3(a)). Compared to room air, the mRNA and protein levels of BCL-2 were significantly increased in the NBO group after 2 (*p* < 0.01, *p* < 0.001) and 6 hours (*p* < 0.05; Figures 3(b) and 3(c)) of reperfusion. Stroke increased the mRNA and protein expression of BAX under room air after 2 and 6 hours of reperfusion, while under NBO, an increase in BAX was significantly inhibited (*p* < 0.05; Figures 3(d) and 3(e)) (see Figure 3).

### 3.5. NBO Induced Neuroprotection through Autophagy Inhibition

**Figure 4:**
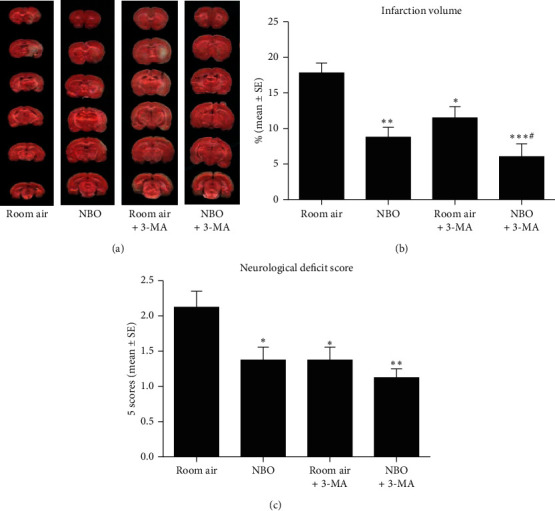
The neuroprotection of NBO was proved to be related to the inhibition of autophagy. (a, b) Brain sections by TTC staining: red for normal tissue and white for infarcted tissue. Compared to room air, 3-MA or NBO reduced infarct volume (*p* < 0.05, *p* < 0.01). (c) Neurological deficit score: the 5-scoring system was used to assess nerve function. Compared to the room air group, NBO, 3-MA, and NBO+3-MA significantly decreased neurological deficit score (*p* < 0.05; *p* < 0.01). ^*∗*^*p* < 0.05, and ^*∗∗*^*p* < 0.01 vs. room air. ^#^*p* < 0.05, vs. room air +3-MA.

To further address the relationship between autophagy and NBO-induced neuroprotection, an intracerebral ventricle injection of 3-MA, the autophagy inhibitor, was applied to the ischemic rats at 6 hours of reperfusion. In comparison with the room air group, 3-MA and NBO both reduced infarct volume (*p* < 0.05, *p* < 0.01; Figures 4(a) and 4(b)), while NBO combined with 3-MA did not further significantly reduce postischemic infarct size. Treatment with NBO, 3-MA, and NBO+3-MA all significantly decreased stroke-induced neurobehavior damage compared to the room air rats (*p* < 0.05, *p* < 0.01; Figure 4(c)). These results indicate that 3-MA did not enhance NBO-decreased ischemic injury after reperfusion, suggesting that NBO may confer neuroprotection by inhibiting the autophagy process (see Figure 4).

**Figure 5:**
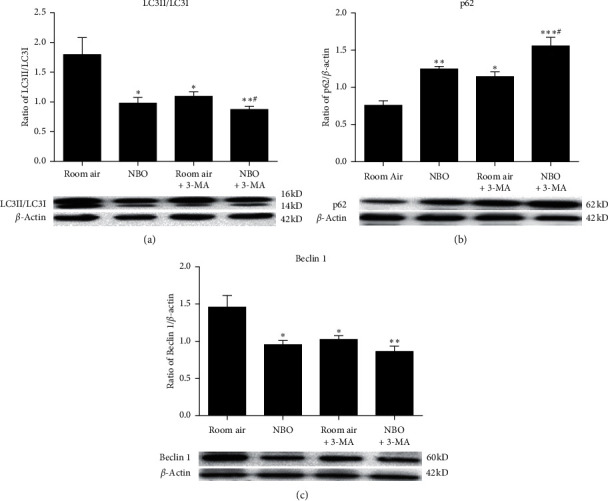
NBO attenuated autophagy activation following tMCAO. (a–c) Western blot and quantitative evaluation of LC3, p62, and Beclin 1 expression. Compared with the room air group, NBO, 3-MA, and NBO+3-MA inhibited the upregulation of LC3 and Beclin 1, with the promotion of p62 level (*p* < 0.05; *p* < 0.01; *p* < 0.001). ^*∗*^*p* < 0.05, ^*∗∗*^*p* < 0.01, and ^*∗∗∗*^*p* < 0.01 vs. room air. ^#^*p* < 0.05, vs. room air +3-MA.

At the protein level, NBO, 3-MA, and NBO+3-MA significantly decreased the expression of LC3II/LC3I and Beclin 1 (*p* < 0.05, *p* < 0.01; Figures 5(a) and 5(c)) and increased p62 expression, respectively, as compared to room air at 6 hours of reperfusion (*p* < 0.05, *p* < 0.01, *p* < 0.001; Figure 5(b)). Again, a combination of NBO with 3-MA did not further reduce autophagy. Together, these results suggest that NBO may have an inhibitory effect on autophagy activation after ischemia/reperfusion (see Figure 5).

### 3.6. Effect of NBO on Apoptosis

**Figure 6:**
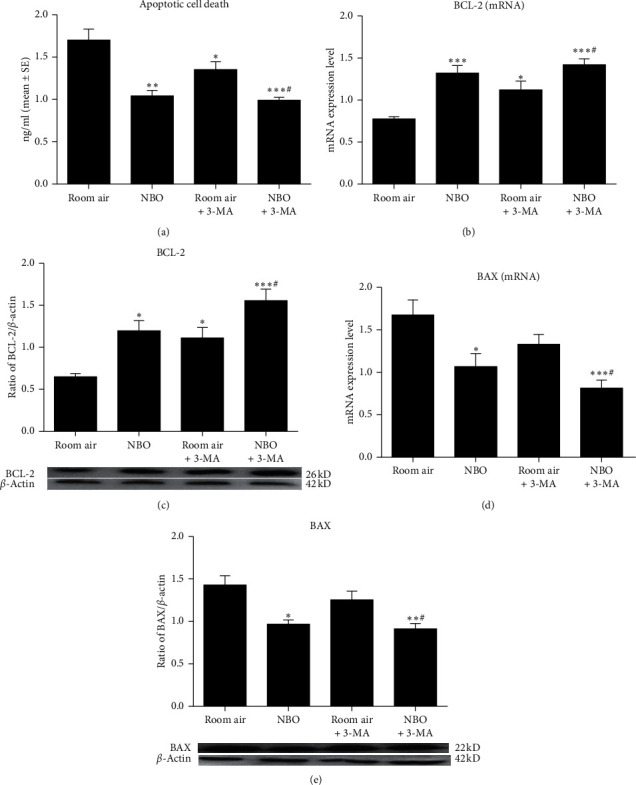
NBO inhibits apoptosis via inhibition of autophagy. (a) Quantitative evaluation of cell death level measured by ELISA kit. NBO and 3-MA reduced cerebral apoptosis levels compared to the room air group (*p* < 0.05; *p* < 0.01). (b, c) 3-MA or NBO significantly increased BCL-2 level at both protein and gene levels (*p* < 0.05; *p* < 0.01). (d, e) NBO inhibited mRNA and protein expression of BAX compared to room air group (*p* < 0.05; *p* < 0.01; *p* < 0.001). ^*∗*^*p* < 0.05, ^*∗∗*^*p* < 0.01, and ^*∗∗∗*^*p* < 0.01, vs. room air. ^#^*p* < 0.05, vs. room air +3-MA.

After 6 hours of reperfusion, NBO and 3-MA both showed a significant reduction in apoptosis and an increase in antiapoptotic BCL-2 expression, respectively, compared to the room air group (*p* < 0.05, *p* < 0.01, *p* < 0.001; Figures 6(a)–6(c)). A significant decrease in proapoptotic BAX expression was seen after the application of NBO (*p* < 0.05, *p* < 0.01, *p* < 0.001; Figures 6(d) and 6(e)). 3-MA with NBO did not further inhibit apoptosis as compared to NBO alone after ischemia/reperfusion. These results suggest that NBO and 3-MA both influence the apoptotic process, and the antiapoptotic effect of NBO after ischemia/reperfusion may be related to the suppression of excessive autophagy (see Figure 6).

## 4. Discussion

In this study, we demonstrated that NBO, given immediately at the beginning of reperfusion, significantly reduced brain infarction, apoptosis, and neurobehavioral deficits after ischemia. Furthermore, as evidenced by the detection of autophagy-related protein expression of LC3, p62, and Beclin 1, NBO inhibited autophagy as early as after 2 hours of reperfusion and up to 6 hours of reperfusion. Our findings suggest that early neuroprotection provided by NBO therapy may be due to attenuation of autophagy that is overactivated by ischemia and reperfusion.

NBO therapy has been considered to have great potential for clinical application in stroke due to its simple, rapid, and noninvasive characteristics. A systematic analysis of previous randomized controlled clinical trial studies found no clear evidence that NBO provides a therapeutic benefit for treating ischemic stroke [31]. Importantly, a recent study by us showed that in patients with anterior circulation cerebral infarction with mechanical thrombectomy, NBO therapy reduced infarct volume and mortality at 3 months without significantly increasing serious complications [12], suggesting that NBO could be a promising therapy for future clinical studies. Several preclinical studies from our group also demonstrated the neuroprotective effect and potential mechanisms of NBO on ischemic stroke. Exposure to normobaric hyperoxia for 6 hours in permanent ischemic rats reduced brain infarct, confirming the neuroprotection of NBO without recanalization [32]. Although NBO treatment has been thought to induce oxidative stress and even aggravate reperfusion injury, Geng's data revealed that administration of NBO for 2 hours immediately after recanalization inhibited reactive oxygen species (ROS) production [33]. Moreover, Ji's study confirmed that t-PA combined with NBO therapy significantly reduced apoptosis in an autologous embolus rat model [34]. In addition, NBO significantly reduced ischemia-induced glucose metabolism disorders based on recanalization [35], which indicates the neuroprotective efficacy of NBO application after ischemia/reperfusion. In the present study, decreased infarct volume and neurological deficits confirmed the protective effect of NBO on the MCAO model, which provides more evidence for the administration of NBO in stroke immediately after recanalization.

The underlying mechanism of neuroprotection by NBO has been thought to be associated with reducing oxidative stress, protecting the integrity of the blood-brain barrier, and improving glucose and lactic acid metabolism [35–38]. Previous neuroprotective therapies have been derived from regulating autophagy after ischemia/reperfusion [39–41]. Autophagy plays a role in oxygen therapy's mechanism of action [24, 25, 42]. Little is known about the influence of autophagy in NBO therapy's mechanism of action. Tian et al. showed that autophagy activation of neurons in the ischemic core and penumbra reached a peak one day after reperfusion of tMCAO [43]. During the process of autophagy, amino acid proteins at the C-terminal of precursor LC3 are degraded to form LC3-I. LC3-I conjugates with phosphatidylserine (PE) to form LC3II, a reliable protein marker for autophagosome formation. p62, another autophagy marker, is recruited into autophagy by LC3 and degraded by the autophagy system. Beclin 1 is involved with phagophore elongation during autophagy [44, 45]. In our research, LC3II/LC3I and Beclin 1 increased significantly in cerebral ischemic damage but decreased after treatment with NBO at 2 and 6 hours of ischemia/reperfusion. Moreover, NBO increased the expression of p62 compared to the expression of p62 on room air during reperfusion after ischemia. This suggests that NBO inhibited ischemia/reperfusion-induced early autophagy. Sole application of the autophagy antagonist 3-MA inhibited the activation of autophagy after ischemia and exerted neuroprotection similar to that of NBO. The combined treatment of 3-MA and NBO did not enhance the effect of NBO. These data together suggest that the reduction of early autophagy activity by NBO contributed to alleviating ischemic injury. It is reported that excessive autophagic activity mediates ischemic injury. Increases in autophagosomes and autolysosomes in injured cortical neurons indicate active autophagy. Neuroprotective therapies targeted toward regulating autophagy include 3-MA, which blocks autophagy with reduced brain edema and motor deficits [18]. Electroacupuncture-induced neuroprotection, another regulator of autophagy, was reversed by the autophagy agonist rapamycin [46]. Furthermore, Huang et al. demonstrated that curcumin induced a significant reduction in LC3 positive cells in stroke rats [47]. Consistent with these studies and neuroprotective treatments, our data, for the first time to our knowledge, found that NBO counteracted the early prodeath mechanism caused by active autophagy after postischemic brain injury.

In addition to autophagy, previous studies have also shown apoptosis in the ischemic penumbra after cerebral ischemia [48]. As a type I programmed cell death, apoptosis regulates the fate of neurons under ischemic conditions. Our results show that NBO reduced neuronal apoptosis after early ischemia/reperfusion by promoting the expression of antiapoptotic protein BCL-2 and inhibiting BAX, a proapoptotic protein [15]. Since apoptosis and autophagy are two death mechanisms that mediate cell fate, emphasis has been placed upon linking the complex regulation between the two [49]. Compound K preconditioning decreased apoptosis after oxygen and glucose deprivation/reperfusion via inhibiting accelerated autophagy [20]. There have also been reported links combining autophagy and apoptosis through interactions of specific proteins between the two processes [50]. Beclin 1, is essential in autophagy initiation to form the autophagic vesicle. It has been confirmed that Beclin 1 has a BH3 domain which could interact with the BCL-2 family of proteins thereby counteracting the antiapoptotic effect of BCL-2 and inducing apoptosis [51]. Gao et al. suggested that BCL-2-mediated apoptosis may be associated with a decrease in autophagic activity after IPOC [26]. Therefore, we hypothesized that the underlying mechanism of antiapoptosis of NBO may be associated with the inhibition of autophagy. Our data revealed that the application of 3-MA and NBO, respectively, interfered with the occurrence of apoptosis after ischemia by decreasing apoptosis and promoting BCL-2 expression, suggesting that autophagy may induce apoptosis under ischemia stimulation manners, but the prodeath mechanism can be reversed by NBO.

There are some limitations to this experiment. First, NBO treatment was only observed in short-term ischemia/reperfusion injury. The long-term efficacy of the treatment will be further determined in future studies. Secondly, in the present study, only one exposure time and concentration of NBO treatment were determined. Variable doses of NBO may provide more information for future clinical and mechanistic investigations. Finally, the specific pathways by which NBO regulates autophagy and the relationship between autophagy and apoptosis are still unclear and warrant further research.

In summary, NBO is a viable treatment after stroke-induced ischemia and reperfusion injury due to its neuroprotective effect, which occurs through intervening with autophagy.

## Data Availability

All original data in this manuscript are available from the corresponding author on reasonable request.
